# The phenotypic and genetic effects of drought-induced stress on wood specific conductivity and anatomical properties in white spruce seedlings, and relationships with growth and wood density

**DOI:** 10.3389/fpls.2023.1297314

**Published:** 2023-12-22

**Authors:** André Soro, Patrick Lenz, Jean-Romain Roussel, Simon Nadeau, David Pothier, Jean Bousquet, Alexis Achim

**Affiliations:** ^1^ Renewable Materials Research Centre, Department of Wood and Forest Sciences, Université Laval, Québec, QC, Canada; ^2^ Canada Research Chair in Forest Genomics, Forest Research Centre and Institute for Systems and Integrative Biology, Université Laval, Québec QC, Canada; ^3^ Natural Resources Canada, Canadian Wood Fibre Centre, Québec, QC, Canada

**Keywords:** xylem specific hydraulic conductivity, conifer, drought-induced stress, heritability, phenotypic and genetic correlations, tracheids

## Abstract

Drought frequency and intensity are projected to increase with climate change, thus amplifying stress on forest trees. Resilience to drought implicates physiological traits such as xylem conductivity and wood anatomical traits, which are related to growth and wood density. Integrating drought-stress response traits at the juvenile stage into breeding criteria could help promote the survival of planted seedlings under current and future climate and thus, improve plantation success. We assessed in greenhouse the influence of drought-induced stress on 600 two-year-old white spruce (*Picea glauca*) seedlings from 25 clonal lines after two consecutive growing seasons. Three levels of drought-induced stress were applied: control, moderate and severe. Seedlings were also planted at a 45° angle to clearly separate compression from normal wood. We looked at the phenotypic and genetic effects of drought stress on xylem specific hydraulic conductivity, lumen diameter, tracheid diameter and length, and the number of pits per tracheid in the normal wood. We detected no significant effects of drought stress except for tracheid length, which decreased with increasing drought stress. We found low to high estimates of trait heritability, which generally decreased with increasing drought stress. Genetic correlations were higher than phenotypic correlations for all treatments. Specific conductivity was genetically highly correlated positively with lumen diameter and tracheid length under all treatments. Tracheid length and diameter were always negatively correlated genetically, indicating a trade-off in resource allocation. Moderate to high genetic correlations sometimes in opposite direction were observed between physico-anatomical and productivity traits, also indicating trade-offs. A large variation was observed among clones for all physico-anatomical traits, but clonal ranks were generally stable between control and drought-induced treatments. Our results indicate the possibility of early screening of genetic material for desirable wood anatomical attributes under normal growing conditions, thus allowing to improve the drought resilience of young trees.

## Introduction

Because of climate change, an increase in the frequency and intensity of heat waves and drought episodes is anticipated in boreal forests (Shukla et al., 2019). Consequently, drought-induced forest decline has emerged as a concern for these forests (Price et al., 2013). Anatomical adaptations of the xylem of trees in response to increasing summer temperatures are generally interpreted as a signal of drought-induced stress (Barber et al., 2000). Understanding how drought-induced stress could affect the wood anatomical properties of ecologically and commercially important species such as white spruce (*Picea glauca* [Moench] Voss) is imperative to better understand how young conifer trees respond to drought stress in the context of intensifying climate change, and delineate more precisely the relationships between wood anatomical traits, seedling growth and wood density.

White spruce is one of the most common and widely distributed temperate-boreal forest tree species in North America, comprising roughly a quarter of the Canadian forest inventory (Canadian Council of Forest Ministers, 2020). It is also highly reforested in various silvicultural systems across the temperate-boreal forests of North America (Nienstaedt and Zasada, 1990), and genetic improvement programs for this species are among the most advanced in Canada (Mullin et al., 2011). Drought-induced stress is known to limit tree growth and water conductivity in this species (Peng et al., 2011; Depardieu et al., 2020; Laverdière et al., 2022; Soro et al., 2022). Drought stress is also the most common cause of mortality in planted tree seedlings (McDowell et al., 2008). D’Orangeville et al. (2018) indicated that white spruce is among the most sensitive to climatic extremes among boreal tree species. Furthermore, following an experience with drought-induced stress treatments in a greenhouse on white spruce seedlings, Soro et al. (2023) observed a decrease in apical and radial growth with the intensity of drought-stress treatment. To identify conifer trees that are best adapted to drought stress at the juvenile stage, including optimal water conductivity in order to maximize survival and growth, it is important to understand the genetic control of the anatomic response of the juvenile xylem to drought-induced stress and how this impacts other traits such as growth.

Xylem responses to drought and heat stress are numerous but can be generally categorized as long- and short-term responses. Trees adapt their water transport pathway both on a seasonal and inter-annual basis (Fonti et al., 2010). Tracheid cell size and shape as well as cell wall thickness can be modified as a response to reduced soil water availability and higher evaporative demand (Rodriguez-Zaccaro and Groover, 2019). Such adaptations can lead to reductions of xylem conductivity to reduce the physiological risks and the impacts of xylem embolism (Gartner, 1995; Rosner et al., 2006). High transpiration during drought increases water tensions in the xylem together with the risk of embolism and dysfunction of the conducting system (Crombie et al., 1985; Sperry and Tyree, 1988). Xylem embolism is recognized as an important physical phenomenon that places unambiguous limitations on water transport (Tyree and Ewers, 1991; Sperry and Saliendra, 1994). Trees may respond to drought through changes in hydraulic traits that can help maintain a favorable water balance (Tyree and Ewers, 1991). For example, prolonged soil drying can cause an increase in the ratio between xylem conductivity and transpiring leaf area (Hacke et al., 2000; Cinnirella et al., 2002). Xylem specific conductivity (ks), which can be used to determine the percentage loss of hydraulic conductance (PLC) or P50 (water potential where 50% of PLC occurs), is the ratio of the flow of water to the pressure gradient responsible for this flow, per unit area of xylem (Tyree and Ewers, 1991) such as cm^2^.

There is a potential trade-off between specific hydraulic efficiency and xylem safety (Linton et al., 1998; Hacke et al., 2000). Indeed, there is a tendency for plants that experience more embolism-inducing stress to build more embolism-resistant xylem, and vice versa (Hacke et al., 2000). Under such conditions, this safety response of xylem to drought stress can negatively influence tree growth and wood properties.

The hydraulic conductivity of the xylem in coniferous species has been estimated using Poiseuille’s law (Zimmermann, 1971). This law describes the laminar flow of a viscous liquid in a cylindrical pipe and its application to a tree implies that tracheids are considered as tubes. This implies that the only source of resistance to the flow of water is the wall of the tracheids. However, in conifers, the sap must also be conducted through the pits of the tracheids (Bailey and Tupper, 1918). Indeed, several early studies indicated that sap flow in conifers could be affected by the number of pits per tracheid (Comstock, 1970; Meyer, 1971; Pothier et al., 1989) and the size of pits (Rosner et al., 2007). The maximum conductivity was shown to increase with the number of pits per tracheid (Aumann and Ford, 2006; Rosner et al., 2007). Hydraulic conductivity was also shown to increase with tracheid length (Aumann and Ford, 2006), and smaller and thicker tracheids were observed under dryer site conditions, together with reduced conductivity (DeSoto et al., 2011). In addition, Rosner et al. (2007) reported that tracheid length, frequency and size of pits per tracheid were under moderate to high genetic control in Norway spruce (*Picea abies* [L.] Karst.).

It thus appears possible to select for anatomical traits that will increase survival and performance of seedlings under drought stress. In a previous study, we showed that the radial growth of white spruce seedlings was much more severely impeded than apical growth by drought-induced stress, which would likely implicate changes in wood anatomy (Soro et al., 2022). However, to our knowledge, no study has yet investigated the relationship between growth and wood anatomical properties likely to be affected by drought stress in the context of selection of more drought-resilient plantation stock such as in white spruce.

In this study, we evaluated the response of wood anatomical features of white spruce clonal seedings submitted to drought-stress conditions and their relationships with growth and wood density traits. We observed the effects of drought-induced stress on wood traits such as tracheid size and number of pits per tracheid. The newly-generated wood anatomical trait data used in this study were obtained from the same seedlings used previously in the drought-stress experiment of Soro et al. (2023), which mainly aimed at monitoring their growth response to such stress. Another objective of the present study was to estimate the amplitude of genetic control in the response of wood anatomical features and specific hydraulic conductivity to drought stress in such young material. By subjecting white spruce seedlings to different levels of greenhouse watering, we assessed: (1) the impact of drought stress on lumen diameter and specific hydraulic conductivity as assessed using Poiseuille’s law, (2) the impact of drought stress on the length and diameter of the tracheids as well as the number of pits per tracheid, (3) the heritability of these various traits and their phenotypic and genetic correlations with growth, wood density and biomass traits at the juvenile stage, (4) the stability of rankings of clonal values under normal and drought-stress conditions, to examine the possibility of indirect selection under normal conditions for improved adaptation to drought stress.

## Materials and methods

### Genetic material and seedling production

The white spruce clonal seedlings used in this study are the same as those described by Soro et al. (2023). The material originates from seed from the best direct crosses of the first breeding cycle, which are multiplied by somatic embryogenesis in order to benefit from the genetic gain resulting from additive as well as non-additive genetic effects (Perron et al., 2018). Seedlings were produced under standard nursery cultural practices at the MFFP forest tree nursery of Saint-Modeste, Québec, Canada. For more details on the genetic material, see Soro et al. (2023).

### Experimental design

The experimental design was described in detail by Soro et al. (2023). Twenty-four seedlings for each of 25 white spruce clones from 19 full-sib families were raised, for a total of 600 seedlings. They were two-year old at the beginning of the experiment, which lasted for two growing seasons and was conducted in a greenhouse at Université Laval, Québec Canada. Knowing that the wood produced during the first years of a tree’s life contains what is called “flexure wood” (Telewski, 1989), the seedlings were planted at an angle of 45° following a similar strategy to that used by Apiolaza et al. (2011) to ensure the production of normal wood. The use of this strategy made it possible to restrict the production of compression wood to only one side of the stems and normal wood otherwise, which was used for our measurements and analyses. All the measurements and data collection for this study were made after the second growing season of drought-induced treatments administered during these two growing seasons. Only the second growth ring was considered in this study, i.e. the ring of the last growing season under drought-induced treatments. As revealed in the previous study of Soro et al. (2023), a modest effect of drought-induced stress on growth and wood density traits was observed after the first year of the experiment, compared to a higher level of effect after two years of drought-stress treatments. Therefore and given the many hundred seedlings under investigation, we opted to collect and analyze data of wood anatomical features only from the last wood ring of the seedlings, thus after two growing seasons under drought-stress conditions. To determine optimal humidity levels, we first determined the field capacity of the substrate by measuring the saturated mass and dry mass. From the dry mass, the volume of water to add to reach the desired humidity level for each of the treatments was determined. The estimation of the optimal substrate water content was based on previous studies which showed that the optimal soil water content for spruce growth is between 60 and 80% of the maximum capillary capacity (Kutílek and Nielsn, 1994; Tužinský, 2002). Another similar study on Norway spruce used 60–80% of maximum capillary capacity for the control treatment, 40–59% of maximum capillary capacity for mild drought stress, and 20–39% of maximum capillary capacity for severe drought stress (Ditmarová et al., 2010). As detailed in Soro et al. (2023), the control treatment was watered (80 to 100% of the maximum capillary capacity) to avoid drought-induced stress while the other two treatments consisted of moderate and severe drought stress. For the moderate drought stress treatment, the substrate was maintained at an intermediate humidity i.e. between 40 and 50% of the substrate maximum capillary capacity. For the severe drought stress treatment, it was decreased to between 20 and 30% of the maximum capillary capacity of the substrate. During the two growing seasons, the light time per day was set to 16 hours in the greenhouse while temperature was set to 23°C during the day and 19°C at night. Three seedlings of one clone (#208) submitted to the severe drought stress treatment died during the second growing season. The 600 clonal seedlings from 25 clones were distributed evenly among the three treatments each replicated twice, for a total of 3 seedlings per clone per treatment per block. For more details on the replicated experimental design, see Soro et al. (2023).

### Measurement of lumen diameter and estimation of specific conductivity

For each seedling after the second growing season, the mean lumen diameter was measured from anatomical cross-sectional images analyzed using the Wincell software (Regent Instruments^®^) (Scholz et al., 2013). The anatomical cross-sections were taken at the same height at the collar level for all young trees. Pictures were taken using a camera connected to a microscope at 400x magnification. A picture was taken of each ring and then analyzed using the WinCell software to estimate the lumen diameters. Theoretical specific conductivity (*k_s_*, kg cm^−1^ MPa^−1^ s^−1^) was calculated for each ring using the modified Hagen–Poiseuille equation described by Tyree and Ewers (1991) and adapted by Wilkinson et al. (2015):


(1) $${\text{k}}_{\text{s}}=\text{N}\frac{\text{π}.{\text{ρ}}_{\text{w}}}{128.\text{η}}\;\frac{1}{\text{n}}\sum_{\text{i}=1}^{\text{n}}\text{L}{\text{D}}_{i}^{4}$$

where *N* (cm^−2^) is the tracheid density of the ring, *n* is the lumen number, *LD* is lumen diameter (cm), *ρ_w_* is the fluid density (assumed to be 1 kg dm^−3^ or equal to that of water at 20°C) and *η* is the fluid viscosity (assumed to be equal to that of water at 20°C, i.e. 10^−3^ Pa·s). We chose to estimate the specific conductivity at the scale of the centimeter (cm) and not of the meter (m) as in the original formula because our data were measured from anatomical sections of seedlings. The scale of the samples being small, we thus used a more appropriate scale.

It should be emphasized that the specific conductivity estimated with **Equation 1** is a theoretical value that makes assumptions and simplifications on the shape and organization of the hydrophilic elements. For example, it does not consider the fact that the characteristics of tracheid pits and tracheids length also influence the flow of water (Pothier et al., 1989).

### Measurement of tracheid size and number of pits per tracheid

**Figure 1:**
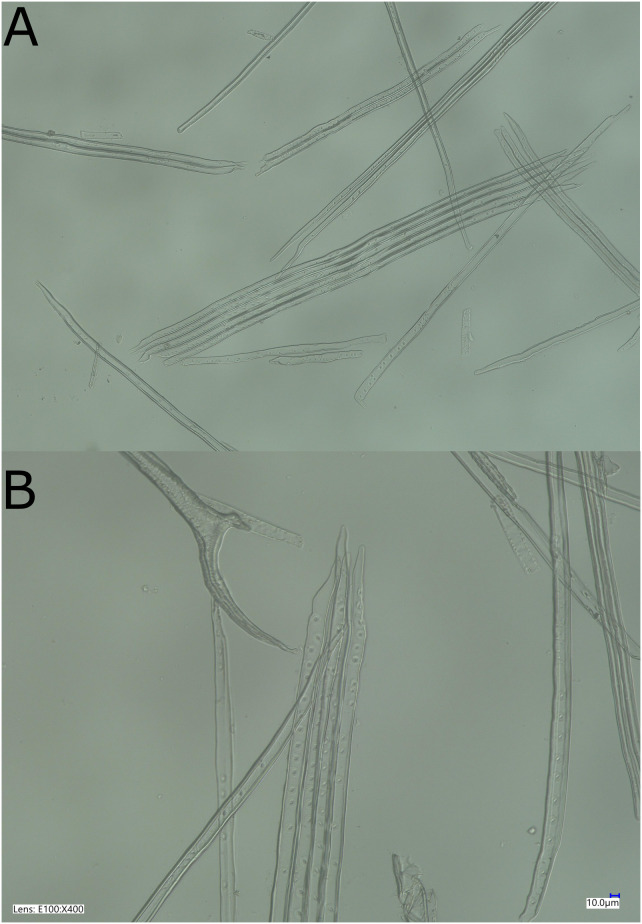
Samples of macerated white spruce wood showing tracheids and pits. The tracheid samples shown here were taken from the same tree. **(A)** Whole tracheids and **(B)** tracheids zoomed on the pits. The reference bar represents 10.0µm. The picture was taken on a Keyence VHX-700 digital microscope.

The length and diameter of tracheids were estimated after wood maceration and using the Fiber Quality Analyzer 360 (FQA) from OpTest Equipment Inc. 1 cm sample was taken from the base of the trunk of each seedling. We thus compared clones and treatments with tracheids measured at the same height at the collar level across all young trees, to avoid any estimation bias that could originate from the position at which the tracheids would have been measured. Samples were first saturated with water by successive cycles of vacuum and pressure. After saturation, samples were immersed in Franklin’s liquor then maintained at 65°C for 48 hours until wood turned white. We subsequently added beads and agitated them to separate the tracheids. Part of the solution was then extracted using a micropipette and poured into a beaker containing about 600 ml of distilled water, which was then stirred. Analyses were the performed using the FQA. Measurements were made on 5000 tracheids for each sample. To estimate the number of pits, we used a randomly selected sample of ten tracheids per seedling on three seedlings per clone for each treatment. We counted the number of pits in each tracheid manually, through observations of macerated wood solution under the microscope. **Figure 1** shows an example of the macerated wood solution in which tracheids and pits are visible.

### Measurement of apical growth, ring width, wood density and biomass index

Annual apical growth increment after the second growing season was measured using a graduated ruler, which should represent the compounded effects of drought stress on apical growth after the two growing seasons. At the end of the experiment, all the seedlings were cut at the level of the collar in order to extract a sample of 2 cm from the widest part of the stem. Similarly as for apical growth, the width of tree rings from the second growing season was then measured, using an optical microscope. The ring width measurements were made in the direction of normal wood, i.e. opposed to compression wood. Wood density was estimated from lumen and cell wall diameter, which were measured from 30 µm thick stem sections using the Wincell software. Sections were stained with safranin and images were taken using a tethered camera under a microscope at 400× magnification. The images were then analyzed using the WinCell software (Regent Instruments^®^) to estimate the proportion of cell wall per unit area. The biomass index was estimated from the estimated wood density multiplied by ring area (Soro et al., 2023).

### Statistical analysis

The open-source R software (R Core Team, 2022) was used for all the statistical analyses and graphic representations. We used linear mixed-effects models to assess the impacts of treatments using treatments and clones as fixed effects and block as a random effect using the lme4 package (Bates et al., 2015). The significance of the difference between treatments was assessed using Tukey’s HSD tests, with *P*< 0.05 as the minimum statistical significance threshold.

### Genetic analysis

Narrow-sense and broad-sense heritabilities, as well as breeding and genetic values of clonal material as expressed by Best Linear Unbiased Predictions (BLUPs), were estimated for each trait with mixed-effects models using ASReml-R v.4.0 (Butler et al., 2017). Narrow-sense heritability considers only the additive genetic variance, while broad-sense heritability takes into account all of the genetic variance (additive, dominance, epistasis) with derivation of clonal genetic values.

The models were of the form (**Equation 2**):


(2) $$\text{y}=\text{Xβ}+{\text{Z}}_{1}\text{a}+{\text{Z}}_{2}\text{b}+{\text{Z}}_{3}\text{c}+\text{e}$$

where ***y*** is the phenotype that represents the different analyzed traits, i.e. xylem specific hydraulic conductivity, lumen diameter, tracheid diameter and length, and the number of pits per tracheid; ***β*** represents the vector of fixed treatment effects and the overall mean, ***a*** is the random additive genetic effect nested within treatment, with ***a*** ∼ ***N*** (**0**, ***V_a_*** ⊗ ***A***); ***b*** is the random block effect, $\text{b}∼\text{N}\left(0,\sigma_{b}^{2}{\text{I}}_{\text{b}}\right)$ ; ***c*** is the random clone effect nested in each treatment, which is accounting for the non-additive genetic effects, including dominance and epistasis, with ***c*** ~ ***N*** (**0**, ***V_c_*** ⊗ ***I_c_)***; and ***e*** is the residual term, with ***e*** ∼ ***N*** (**0**, ***R***), where ***R*** is a block diagonal matrix specifying a heterogeneous error variance structure for the three treatments (for more details see Soro et al., 2023). ***A*** is the pedigree-based relationship matrix quantifying relatedness between clones from sharing common parents. ***I_b_*** and ***I_c_*** are identity matrices of their proper dimension. $\hat{V}$ ***_a_*** and $\hat{V}$ ***_c_*** are 3 × 3 variance–covariance matrices defined by the additive and clonal correlations between treatments and unique additive and clonal variances for each treatment, respectively (i.e. CORGH in ASReml-R). The symbol ⊗ refers to the Kronecker product. The matrices **X**, ***Z_1_***, ***Z_2_*** and ***Z_3_*** are incidence matrices of their corresponding effects.

Heritabilities were estimated by the same method used in Soro et al. (2023) (**Equation 3**):


(3) $${\hat{H}}_{ind}^{2}=\frac{\left(\bar{{\hat{\sigma }}_{a}^{2}}+\bar{{\hat{\sigma }}_{c}^{2}}\right)}{\left(\bar{{\hat{\sigma }}_{a}^{2}}+\bar{{\hat{\sigma }}_{c}^{2}}+\bar{{\hat{\sigma }}_{e}^{2}}\right)}$$

where ${\hat{H}}_{ind}^{2}\;$ is the individual broad-sense heritability, $\bar{{\hat{\sigma }}_{a}^{2}}\;$ is the average additive genetic variance across treatments, $\;\bar{{\hat{\sigma }}_{c}^{2}}\;$ is the average non-additive genetic variance, and $\bar{{\hat{\sigma }}_{e}^{2}}$ is the average residual variance. Standard errors of heritability estimates were obtained using the delta method (vpredict function from the ASReml-R, version 4.0; Wolak, 2012).

To estimate phenotypic and genetic correlations between traits for each treatment, bivariate models were fit for all pairs of traits in ASReml-R. The method used is the same as in Soro et al. (2023) (see **Supplementary**).

### Spearman’s rank correlations (Spearman’s ρ)

To assess genotype-by-environment interactions (GxE), meaning in this experiment the interactions between clones and treatments of drought-induced stress, we calculated in a pairwise fashion Spearman’s rank correlation coefficients (ρ) of clonal mean trait values between treatments. The cor.test (stats) function was used for the calculations.

## Results

### Trait phenotypic variation among treatments

**Figure 2:**
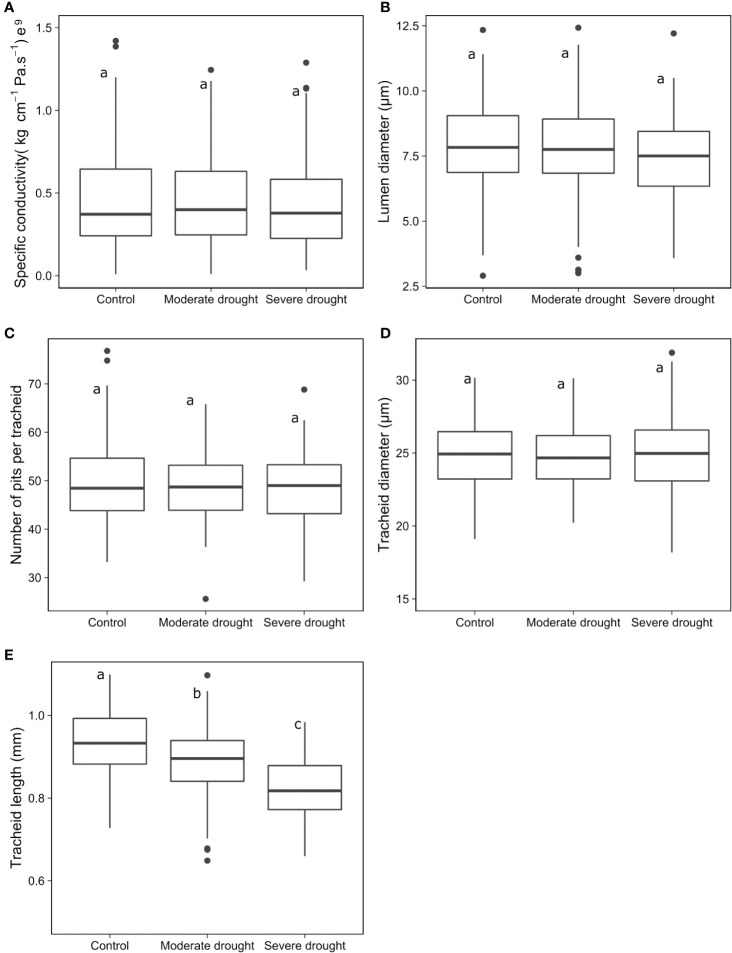
Comparison of tree responses to different levels of drought-induced stress for specific conductivity **(A)**, lumen diameter **(B)**, number of pits per tracheid **(C)** and tracheid diameter **(D)** and length **(E)**. Means with different letters indicate significant differences at *P*< 0.05 (Tukey tests).

**Figure 2** shows the effects of drought-induced stress treatments on each physico-anatomical trait at the phenotypic level. Except for tracheid length, variation among treatments was not statistically significant (*P* > 0.05) for any other trait (specific conductivity, tracheid and lumen diameters, and the number of pits per tracheid), even if some decreasing trends could be observed for some traits (**Figure 2**). Tracheid length sharply decreased with the level of drought intensity (**Figure 2**), which was supported by a statistically significant Tukey test value (*P*< 0.05).

**Figure 3:**
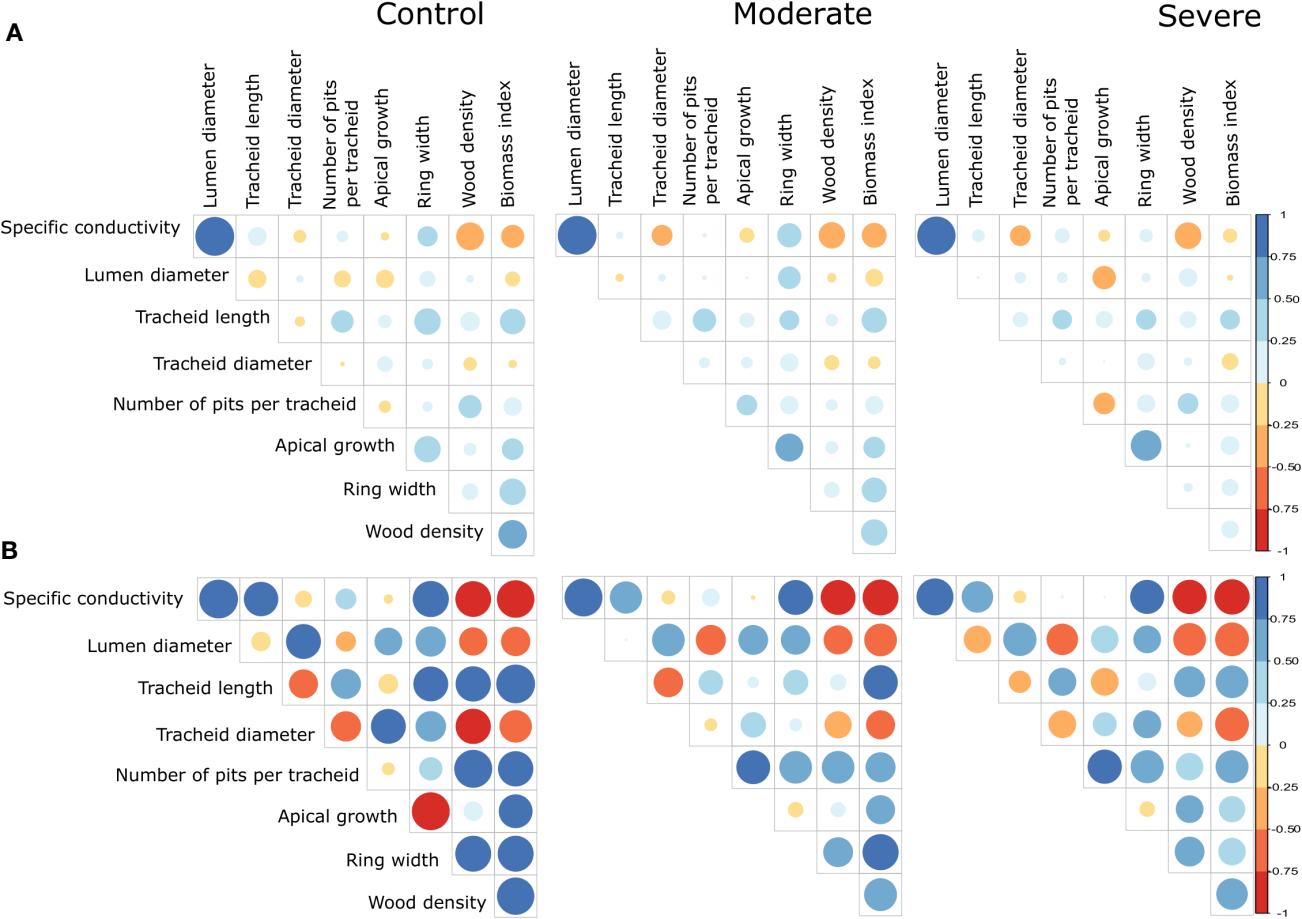
**(A)** Matrices of phenotypic correlations between the different traits for each treatment, i.e. the control, moderate and severe drought stress treatments. **(B)** Matrices of genetic correlations between the different traits for each treatment. The higher the correlation, the larger the size of the dot and intensity of the color. The color gradients indicate the direction and level of correlations (blue for positive and red for negative correlations). Corresponding values and their standard errors can be found in **Supplementary Tables 1** and **2**.

The matrices of phenotypic correlations between traits for each treatment showed mostly weak correlations with a few higher ones (range from -0.36 to 0.99) (**Figure 3A**) (**Supplementary Table 1**). The highest and positive phenotypic (and genetic) correlations were observed between lumen diameter and specific conductivity for all treatments, given the large weight of lumen diameter in Hagen–Poiseuille equation used to estimate specific conductivity (see Materials and Methods, **Equation 1**). Tracheid length also showed moderate positive phenotypic correlations with the number of pits per tracheid, which was also expected. Positive phenotypic correlations were also noted between tracheid length and ring width and biomass index. Otherwise, generally null or weak phenotypic correlations were observed between physico-anatomical traits (specific conductivity, lumen diameter, tracheid length, tracheid diameter and number of pits per tracheid), and between these and productivity traits (apical growth, ring width, wood density and biomass index). Phenotypic correlations between productivity traits were previously reported and discussed (Soro et al., 2023).

Overall, genetic correlations among traits (**Figure 3B**) (**Supplementary Table 2**) were much higher compared with their phenotypic counterparts (**Figure 3A**) (**Supplementary Table 1**), which is a quite notable trend. Thus, we will mostly focus the following description on trends in genetic correlations.

For the majority of traits, a noticeable general decrease in the amplitude of genetic correlations was observed between control and drought-induced conditions, indicating less genetic determinism and thus, more environmental influence on trait expression under drought conditions (**Figure 3B**). Regarding genetic correlations between physico-anatomical traits, high positive values were observed between lumen diameter and specific conductivity, given the large weight of lumen diameter in Hagen-Poiseuille formula to estimate specific conductivity. For all treatments, positive genetic correlations were also observed between specific conductivity and tracheid length. As well, positive genetic correlations were observed between lumen diameter and tracheid diameter, and between tracheid length and number of pits per tracheid, while negative genetic correlations were observed between tracheid diameter and number of pits per tracheid, which conforms to normal expectations given the negative genetic relationships observed for all treatments between tracheid length and diameter. These last negative relationships indicate a clear shift in resource allocation between tracheid length and diameter. Genetic correlations were also negative between lumen or tracheid diameter on one side, and wood density on the other side, which is likely due to increased cell wall thickness and wood density as lumen diameter decreases. On the opposite side, genetic correlations were positive between tracheid length and wood density, reflecting the shift in resource allocation already noted between tracheid length and diameter. Thus, there seems to be a positive relationship, though assessed indirectly, between tracheid length and cell wall thickness, as the genetic correlations were generally positive between tracheid length and wood density.

Between all wood anatomical features and ring width, genetic correlations were moderate to high for all three treatments and, as a result, specific conductivity was also highly correlated to ring width (**Figure 3B**). Regarding the genetic relationships between wood anatomical features and apical growth, they were quite diverse and variable from one treatment to the other, with weak to moderate positive genetic correlations with lumen and tracheid diameters for all treatments, low to moderate negative genetic relationships with tracheid length under the control and severe drought stress treatments, respectively, and noteworthy strong positive genetic correlation with the number of pits per tracheid only under the drought-stress treatments (**Figure 3B**). Accordingly, apical growth was only weakly correlated genetically to specific conductivity for all three levels of drought-stress treatment, contrary to ring width which was positively related to specific conductivity at the genetic level. As seen in **Figure 3B** and as previously reported by Soro et al. (2023), while weakly positive phenotypic correlations were noted between ring width and apical growth for all three treatments, negative genetic correlations were noted, especially for the control treatment, thus indicating a genetically-determined trade-off in resource allocation between radial and apical growth under non stressful conditions, that weakened under drought-stress conditions.

### Linear regression models between growth traits and tracheid length in severe drought-stress conditions

**Figure 4:**
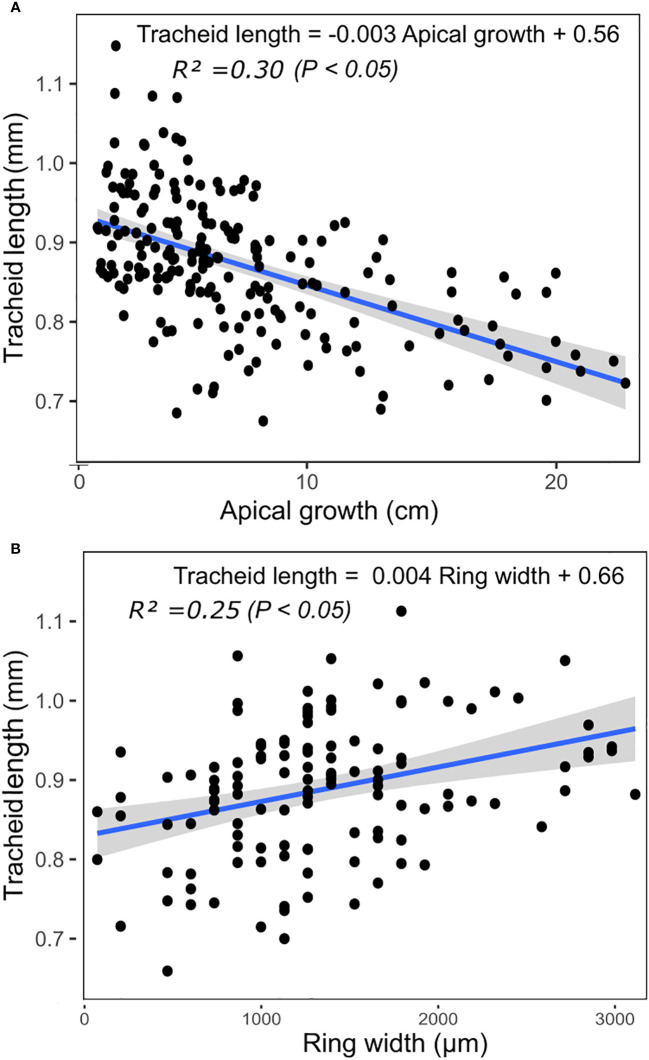
Linear relationships (*P*< 0.05) between tracheid length and apical growth **(A)** and ring width **(B)** under severe drought conditions, at the phenotypic level.

Because of the noticeable decrease in tracheid length with increasing level of drought stress (**Figure 2E**), we investigated more in details the relationships with growth traits in most stressful conditions (**Figure 4**). Under severe drought-stress conditions, we observed a negative linear relationship between tracheid length and apical growth, with a coefficient of determination *R^2^* of 0.30 (*P*< 0.05, **Figure 4**)). Conversely, there was a positive linear relationship between tracheid length and ring width with a *R^2^* of 0.25 (*P*< 0.05, **Figure 4B**). Power functions were also tested and the best ones had a *R^2^* of 0.29 (*P*< 0.05) between tracheid length and apical growth, and *R^2^* of 0.25 (*P*< 0.05) between tracheid length and radial growth.

### Heritability estimates and stability of clonal ranks across treatments

**Figure 5:**
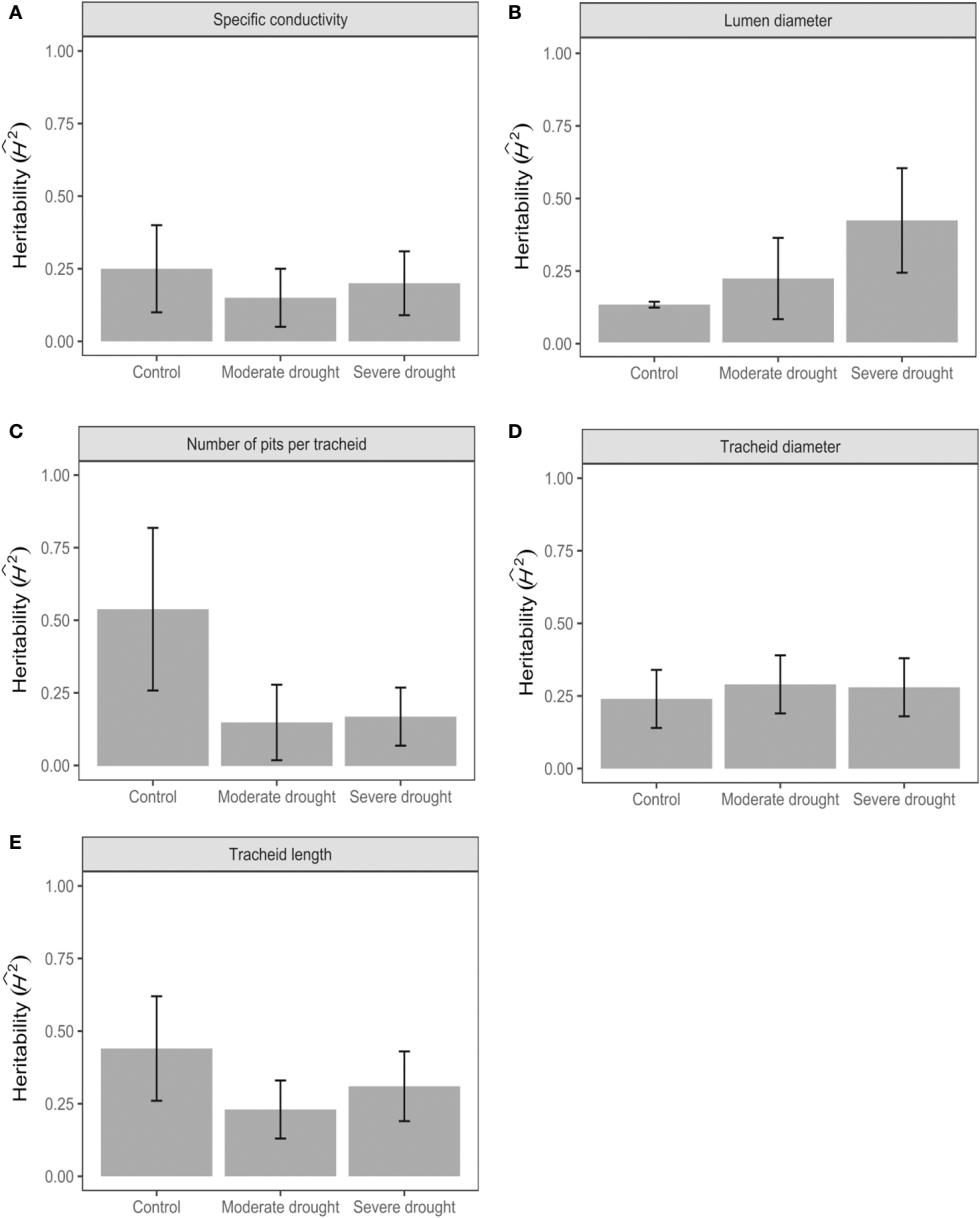
Broad-sense heritability estimates for specific conductivity **(A)**, lumen diameter **(B)**, number of pits per tracheid **(C)**, tracheid diameter **(D)** and tracheid length **(E)**. Error bars represent the standard errors. Narrow-sense heritability values and their standard errors can be found in **Supplementary Table 3**.

Specific hydraulic conductivity, lumen diameter, tracheid length, tracheid diameter, and the number of pits per tracheid had low to high broad-sense heritability (from 0.13 to 0.54) (**Figure 5**) (**Supplementary Table 3**). For most of heritability estimates, the values were above zero from judging by the size of standard errors of estimates. In interpreting qualitatively the amplitude of heritability estimates, we followed conventions where low values are usually those below 0.30, moderate values are those between 0.30 and 0.50, and high values are those equal to or above 0.50. Depending on the physico-anatomical trait, different trends were noted in relation to the level of drought stress treatment.

The highest heritability estimate under control conditions was observed for the number of pits per tracheid, with progressively weaker genetic control with increasing severity of drought-induced stress, thus indicating an increased environmental influence on trait expression with drought-stress level (**Figure 5**). Under severe drought-stress conditions, the highest heritability was observed for lumen diameter, which showed an increasing trend with the intensity of drought-stress conditions beyond standard errors of estimates. This pattern of stronger genetic control under stressful environmental conditions was thus opposite to that observed for the number of pits per tracheid. For the other physico-anatomical traits, patterns were more regular across treatments where slightly different values were observed, but within the range of standard errors of estimates. For instance, quite uniform and moderate values of heritability were observed for tracheid diameter, and a slight loss of heritability under drought-stress conditions was noted for tracheid length and specific conductivity (**Figure 5**).

**Figure 6:**
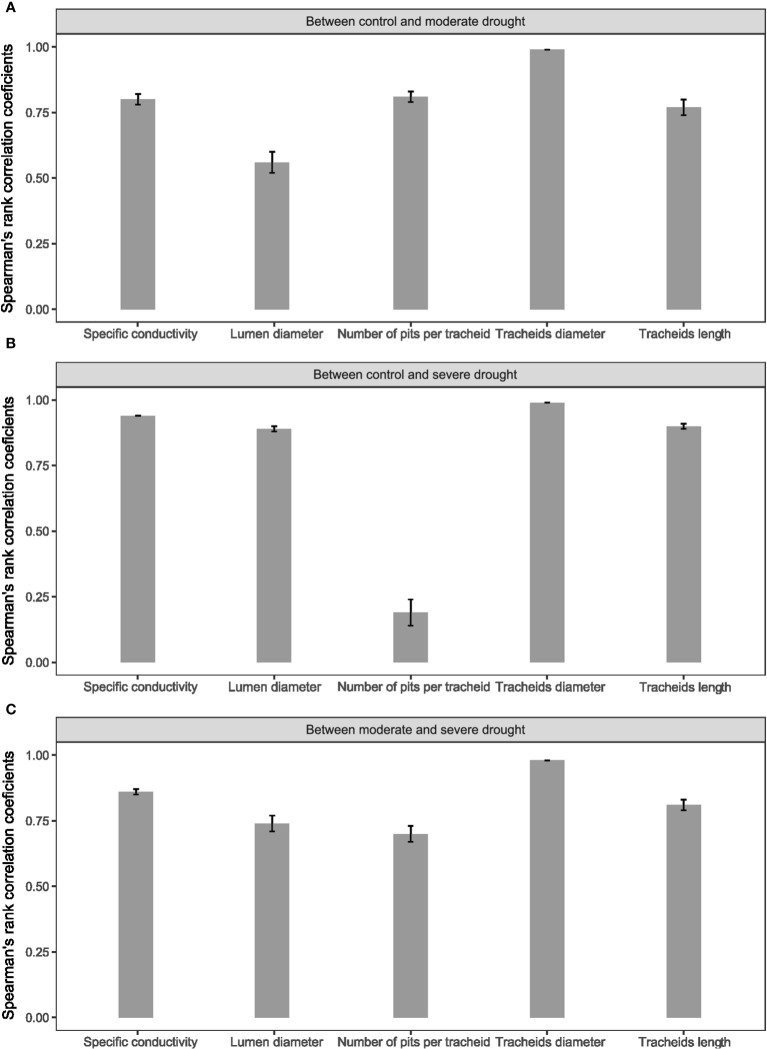
Spearman’s rank-order correlation coefficients of clonal values that consider additive and non-additive genetic effects between treatments, **(A)** control and moderate drought, **(B)** control and severe drought, **(C)** moderate and severe drought, for specific conductivity, lumen diameter, tracheid length, tracheid diameter and the number of pits per tracheid. Error bars represent the standard errors.

In order to assess the relative stability of clone performance across the different drought-stress treatments, we estimated Spearman’s rank-order correlations of clonal values between the different treatments. For specific conductivity, lumen diameter, as well as tracheid diameter and length, Spearman’s correlations were moderate to high (0.56 to 0.99) among all treatments (**Figure 6**). For the number of pits per tracheid, a high correlation was observed between the control and moderate drought-stress treatments (0.80) and between the moderate and the severe drought-stress treatments (0.70), but the correlation was much reduced between the control and severe drought-stress treatments (0.19). This result is likely related to the large decrease in genetic control for this trait under the severe drought-stress treatment compared to control conditions (**Figure 5**). Besides this exception for the number of pits per tracheid, these results indicate that the ranking of the clones for the other physico-anatomical traits remained relatively stable across the different treatments, which is a quite encouraging trend for early selection under normal conditions for increased resilience to drought stress (see Discussion).

## Discussion

### Drought-stress effects on xylem specific conductivity and wood anatomical traits

There was no effect of the level of drought-stress treatment on any trait except for a marked reduction in tracheid length with increasing severity of drought-stress treatment (**Figure 2**). The similitude in the responses of other physico-anatomical traits between the control and drought-induced treatments should be considered as an encouraging result regarding the evaluation of seedling response to drought stress. It indicates that most of the physico-anatomical traits are not severely affected by drought stress at the phenotypic level and that, in general, the evaluation of traits under non-drought conditions could well inform about trait values under non-lethal drought-stress conditions. This general trend was also quite congruent with those observed regarding heritability estimates and stability of clonal values (**Figures 5** and **6**).

As for the observed marked reduction in tracheid length, in particular under severe drought-stress treatment (**Figure 2**), this pattern is in accordance with those of several previous studies including those with trees at the more mature stage, which showed that tracheid length tends to decrease with increasing drought-stress severity (Pothier et al., 1989; Ladjal et al., 2005; Rosner et al., 2007; DeSoto et al., 2011). The net effect of this reduction in tracheid length was associated to a relative increase in apical growth and slight decrease in radial growth under severe drought-stress conditions (**Figures 3** and **4**). A study by Lazzarin et al. (2016) showed that the length of tracheids can vary along the stem from the root collar to the apex. Given that the apical growth of the seedlings was quite affected by drought-stress conditions (Soro et al., 2023), it is possible that the decrease in tracheid length under such conditions, as observed in our study from sampling wood rings at the base of the stems, could represent partly an indirect effect of drought on apical growth, because of the distance progressively increasing from the base of the stem to the apex. Further work with multiple samplings of tracheids along seedling stems submitted to drought-stress treatments or not, would be needed to fully identify and disentangle these possible effects.

Even if a generally moderate positive genetic relationship was observed between tracheid length and the number of pits per tracheid (**Figures 3A, B**), the number of pits per tracheid did not vary much across treatments at the phenotypic level (**Figure 2**). Because of the marked overall reduction in tracheid length under water stress conditions (**Figure 2**), such a moderate increase in the number of pits per tracheid would not compensate for the negative effect of this reduction on radial growth (**Figures 3B** and **4**). Thus, under such severe stress and reservations made above, reduced tracheid length would likely represent a main cause of reduced radial growth and not the number of pits per tracheid, given that shorter tracheids reduce conductivity, as shown by the positive genetic relationships between these two traits (**Figure 3B**), and as indicated in previous studies at the phenotypic level (Meyer, 1971; Aumann and Ford, 2006; DeSoto et al., 2011).

After lumen diameter, tracheid length was also the second most positively correlated anatomical trait with specific hydraulic conductivity at the genetic level (**Figure 3B**), even if it was not implicated in the formula to estimate conductivity (see Materials and Methods) and thus, this correlation could be coincidental. To verify if this correlation could be of causal nature, real conductivity should be assessed. However, in itself, the reduction in the length of tracheids under drought-induced conditions could be considered as an adaptive mechanism of “drought damage avoidance”, because it would contribute towards decreasing the rate of transpiration and reducing the risks of embolism or, alternatively, to contain the embolisms in shorter units. Direct measurements of embolism would help confirm this hypothesis.

Regarding tracheid diameter, it was little affected by the level of drought-induced stress (**Figure 2**), which echoes the findings of Jyske et al. (2010) in Norway spruce and Held et al. (2021) in Scots pine (*Pinus sylvestris*). In contrast, Larson (1963) reported that tracheid diameter tended to increase with drought severity in red pine (*Pinus resinosa* Ait.), which could suggest different mechanisms of drought response at the wood anatomical level between these distantly-related genera of the Pinaceae, whose lineages are separated by more than 120 million years (Savard et al., 1994). As for tracheid length, some studies also showed that tracheid diameter may vary depending on the distance between the sampling location of wood rings and the apex of the stem (Anfodillo et al., 2006; Kiorapostolou et al., 2020). Again, the fact that we did not observe a strong effect of drought-induced stress on tracheid diameter must thus be put into perspective. The effects of drought stress on apical growth may have masked a possible effect on tracheid diameter at a fixed distance from the apex.

Similarly, as indicated above, drought-induced stress conditions did not affect much the number of pits per tracheid. This result is similar to that observed by Rosner et al. (2007) in Norway spruce, but they did not find any relationship between tracheid length and the frequency of pits per tracheid. In our study, we did find a positive relationship both at the phenotypic and (even more so) at the genetic levels between these two tracheid traits (**Figure 3B**). Our results are also in agreement with those of Jinxing (1989) on the more distantly-related Monterey pine (*Pinus radiata* D. Don), who found that the number of pits per tracheid in the first ten years of growth was related more to tracheid length than to cambial age. In the same study, the number of pits per tracheid was mainly related to cambial age beyond the tenth year, regardless of tracheid length. Thus, it is likely that such a relationship between tracheid length and the number of pits per tracheid is age-dependent, given the juvenile nature of the material tested in our study.

No significant effect of drought-induced stress on the estimated specific conductivity of the xylem was observed (**Figure 2**). Given the large dependency of this parameter on lumen diameter, which also did not vary significantly across treatments, this result is not surprising. It echoes the results obtained by Croisé et al. (2001) in Scots pine (*P. sylvestris* L.) seedlings. These authors did not report a significant effect of drought stress on direct measurements of hydraulic conductivity. A possible explanation for the lack of response of specific conductivity to drought-induced conditions in our study could be that the general formula used to estimate specific hydraulic conductivity does not consider the length of tracheids and the number of pits per tracheid, which are potential contributors to the real xylem water conductivity. Poiseuille’s law or Darcy’s law are commonly used to estimate specific hydraulic conductivity under the assumption that tracheids behave as interconnected cylindrical tubes. However, conifer tracheids are connected by pits, and are thus not completely analogous to hydric tubes (Hacke et al., 2004). Considering tracheid structure, the estimation of hydraulic conductivity in softwood stems should likely integrate tracheid length, and perhaps the number of pits per tracheid, so to provide a more realistic assessment of overall hydraulicity. This observation is also supported by empirical evidence from several previous studies (Comstock, 1970; Meyer, 1971; Smith and Banks, 1971; Pothier et al., 1989; Hacke et al., 2004; Aumann and Ford, 2006).

It was also reported that smaller lumen diameter resulting from lower cell and xylem expansion (Bowyer et al., 2005) might lead to greater resistance to embolism during drought episodes (Tyree and Zimmermann, 2002). Though we did not assess directly embolism, lumen diameter was statistically uniform across treatments (**Figure 2**), with a statistically non significant reduction of lumen diameter as drought-stress level increased (**Figure 2**). This predominant trend of uniformity across treatments was also observed for specific conductivity, which was largely positively correlated to lumen diameter at the phenotypic and genetic levels (**Figures 3A, B**), given the large weight of lumen diameter in Hagen-Poiseuille formula used to estimate specific conductivity (Tyree and Ewers, 1991; Wilkinson et al., 2015). In our experiment, drought stress might not have been intense enough to cause a statistically significant reduction in lumen diameter. It is likely than in our white spruce seedling material, reduced tracheid length had the most decisive role in limiting embolism under the most severe drought-induced stress conditions.

### Phenotypic and genetic correlations between physico-anatomical traits

First and foremost, a clear pattern was observed where much higher genetic than phenotypic correlations were observed among physico-anatomical wood traits, and between these and growth traits, wood density and biomass (**Figure 3**). While such a trend was also observed in our previous study of productivity traits (Soro et al., 2023), it indicates that the mechanistic and functional relationships between traits could be better deciphered by disentangling genetic from environmental effects. This trend also indicates that despite care taken to conduct the study in greenhouse controlled conditions and minimizing micro-environmental variation, there was still much environmental effects affecting the phenotypic correlations obtained, contrary to genetic correlations.

A few other conifer genetic studies investigating variation in wood anatomical traits such as tracheid length, and their relationships with growth and wood density traits, also found higher genetic than phenotypic correlations (Zhang and Morgenstern, 1995; Hylen, 1997; Gaspar et al., 2008). Also, we observed higher genetic than phenotypic correlations for all levels of drought-induced stress. This indicates that trait relationships under normal growing conditions can represent a good indirect assessment of relationships under drought-stress conditions, as there was little or no significant reversal of genetic correlation trends under such conditions. In addition, clonal ranks were maintained quite well across all treatments for most physico-anatomical traits including lumen and tracheid diameters, and tracheid length (**Figure 6**). Those are very encouraging results for selection of more resilient spruce juvenile material for reforestation and material that would be less prone to embolism under severe drought-stress conditions at the juvenile stage, given that mechanistic relationships observed under control conditions seem to be maintained quite well even under severe stress conditions.

Lumen and tracheid diameters were found genetically highly positively correlated (**Figure 3B**), which indicates that much of the variation in tracheid diameter could be accounted for by lumen diameter instead of cell wall thickness. This is because even if cell wall thickness was not assessed directly in this study, the genetic correlations were high and negative between these two tracheid diameter traits and wood density across treatments, thus indicating a clear positive effect of lumen diameter on tracheid diameter. At the phenotypic level, no such clear relationships could be observed, indicating the existence of conflicting environmental influence on trait expression.

A clear trade-off was also noted at the genetic level between tracheid length and tracheid diameter, not only for the control treatment but also under drought-induced stress conditions (**Figure 3B**). At the phenotypic level, this trade-off was not significant. Thus, there appears to be a clear resource allocation strategy at the genetic level between these two crucial aspects of tracheid development and morphology. This differential allocation of resources tended to weaken under severe drought conditions, indicating more conflicting physiological signals under such growth-limiting conditions.

High positive genetic correlations were observed between lumen diameter and specific conductivity. This is a normal expected outcome given the large weight of lumen diameter in Hagen-Poiseuille equation used to estimate specific hydraulic conductivity (Tyree and Ewers, 1991; Wilkinson et al., 2015). However, tracheid length was genetically highly positively related to specific conductivity. The number of pits per tracheid followed the same trend but to a lesser extent, even if these two anatomical traits were not considered in Hagen-Poiseuille equation used herein to estimate specific conductivity. Despite this caveat, these tracheid traits (tracheid length, tracheid diameter and number of pits per tracheids) seem to represent key parameters in the seedling response to drought-stress conditions, and they should be better considered in estimating overall conductivity in conifer wood. As observed under drought-stress treatments, shorter tracheids likely contributed to hydraulic safety and resilience to drought stress. One hypothesis is that this would be caused by segmenting of embolism effects into shorter units, which would need to be assessed more directly.

### Genetic relationships between physico-anatomical and productivity traits

At the genetic level, all anatomical traits were positively correlated to ring width across treatments (**Figure 3B**). But regarding apical growth, the patterns were less concerted, especially at the genetic level. Indeed, tracheid length was negatively correlated with apical growth for both phenotypic and genetic relationships (**Figures 3** and **4**), while only tracheid and lumen diameters were positively related to apical growth under all conditions. The number of pits per tracheid became positively associated genetically to apical growth only under moderate to severe drought stress conditions, likely indicating a more crucial role of this anatomical feature under more critical conditions for sap flow. Under severe drought-stress conditions, genetic correlations opposite to phenotypic correlations were generally observed between most anatomical traits and apical growth. (**Figure 3**). These trends indicate the conflicting influences of genetic and environmental factors affecting the relationships between wood anatomical traits and apical growth, which is recognized as a most sensitive growth trait to drought-stress conditions (Soro et al., 2023).

Although embolism could not be directly assessed in this study, the negative genetic correlation observed between tracheid length and apical growth, especially under severe drought-stress conditions, suggest that selection for shorter tracheids would lead to proportionally more apical growth by improving resistance to embolism under severe drought-stress conditions and thus, confining breakage of the water column in shorter units. On the other hand, early selection for longer tracheids by tree breeders is often mentioned as a key aspect for better wood quality at the more mature stage (e.g. Beaulieu, 2003). If so, one hypothesis to be tested is that such selection scheme could likely result in lower resistance to embolism at the juvenile stage and, consequently, reduced apical growth, as observed in our material under severe drought-stress conditions.

Given the context of climate change and tree breeding material facing more extreme environmental conditions of various nature, it thus appears that clones with reduced tracheid length at the juvenile stage would show superior apical growth under severe drought-stress conditions, given that they would be more resilient to drought stress. This would likely confer higher survival rate and better competitive advantage in the context of reforestation. Long-term follow-up studies monitoring at different ages the changes in rankings of the clones regarding tracheid length, in order to estimate the strength of age-age correlations, would also be needed to assess any detrimental effect of such juvenile selection for better resilience to drought stress, on tracheid length and wood quality at the more mature stage.

While lumen diameter and tracheid diameter had negative genetic correlations with wood density and biomass index, tracheid length and the number of pits per tracheid followed an opposite trend of positive genetic relationships with these traits (**Figure 3B**), which is in agreement with the results of Klisz (2011) for earlywood density in European larch. However, we observed a weakening of these correlations under drought-stress treatments, indicating more adverse environmental influence under such conditions. Regarding the reduced apical growth under drought-stress conditions, other factors than tracheid length are likely to have contributed to this negative trend, including a more limited development of juvenile root systems leading to significantly reduced growth of seedlings under drought-induced treatments (Soro et al., 2023).

We observed a positive genetic relationship between tracheid length and specific conductivity although tracheid length is not taken into account in the Hagen-Poiseuille formula. Additionally, positive genetic correlations were observed between tracheid length and ring width, wood density and biomass even under drought stress conditions, and it was quite stable among clones across treatments (**Figure 6**). These trends indicate that clonal values of tracheid length under normal conditions could be used as a good proxy for indirect selection under water-stress conditions, together with using specific hydraulic conductivity for the same purpose, whenever it could be estimated. However, if apical growth stands as the priority productivity trait at the juvenile stage, for instance in order to provide a competing advantage for light in reforestation landscapes, larger tracheid diameter under normal conditions would likely represent a better anatomical feature for indirect selection under water-stress conditions (**Figures 3B** and **6**).

Regarding lumen diameter, despite its positive genetic relationships with both apical and radial growth traits across treatments (**Figure 3B**), it had negative genetic correlations with wood density and seedling biomass. This trend is a normal expectation under the well-known genetic trade-off observed in more mature white spruce trees between diameter growth and wood density (Beaulieu, 2003; Lenz et al., 2010). These correlations would further indicate indirectly that lumen diameter was negatively associated to cell wall thickness in our experiment, which could not be assessed directly.

### Tracheid length as a key factor for juvenile growth under severe drought conditions

Tracheid length was positively related to radial growth and negatively related to apical growth (**Figure 4**). This relationship was also clearly observed at the genetic level (**Figure 3B**). It is plausible that by limiting embolism and maintaining hydraulic safety under severe drought-stress conditions, a reduction in tracheid length would result in a relatively more limited radial growth than apical growth. This pattern is supported by the observations of Zobel and Van Buijtenen (1989), who reported that tracheids are usually shorter in narrow rings than in large rings. Others have also reported similar positive phenotypic and genetic correlations between tracheid length and radial growth in more mature spruce trees, including in white spruce (Beaulieu, 2003) and Norway spruce (Hannrup et al., 2004). However, these studies did not indicate if the narrower rings were related to drought stress or other environmental factors.

When resources are limited by severe drought-stress conditions, the allocation of photosynthates may change between radial (ring width) and apical growth, and the prioritization of radial growth may thus be limited by the risk of hydraulic failure. Thus, in addition to wood anatomical features such as tracheid length, other factors of more physiological nature such as embolism may have affected negatively radial growth in such severe drought-induced conditions suffered by the young seedlings during two consecutive growing seasons. This observation is also supported by the noted greater sensitivity of radial growth than apical growth to such severe growing conditions (Soro et al., 2023), and because of the lesser genetic control of radial growth variation than that of apical growth in such detrimental environmental conditions (Soro et al., 2023). Thus, given the negative relationship between tracheid length and apical growth under severe drought-stress conditions, and given the net negative effect of drought stress on apical growth, a general strategy of drought-stress response and avoidance of cellular damage due to severe drought stress appears to be the production of shorter tracheids with reduced hydraulic conductivity but better resistance to embolism, giving rise to proportionally improved apical growth compared to clones with longer tracheids when experiencing severe drought-stress conditions.

### Heritability and stability of clonal responses

All physico-anatomical traits had moderate to high broad-sense heritability values (**Figure 5**), indicating the existence of significant genetic variance when considering both additive and non-additive genetic effects. Notable variation in genetic control was also observed between control and drought-induced conditions for traits such as lumen diameter where heritability was higher under drought-stress conditions, and weaker under such conditions for tracheid length and the number of pits per tracheid. For instance, the much higher genetic control of lumen diameter under severe drought than under normal conditions indicates that juvenile selection for this trait under severe drought conditions could result in higher genetic gains, while the opposite would be true for tracheid length. Such opposite patterns indicate an intricate balance between genetic and environmental factors in the control of trait expression under varying environmental conditions. As such, the reduction in hydraulic vulnerability through modifications of xylem anatomy appears to be much under mixed genetic and environmental influences. Given the juvenile nature of the material tested in this study, it remains difficult to extend these observations to more mature trees. Nevertheless, hydraulic stress is an important cause of mortality in young plantations (McDowell et al., 2008) and thus, it remains important to study the drought-stress response of wood anatomical traits at the seedling stage, especially if juvenile selection is to be deployed in order to increase resistance and survival rate under drought-stress conditions.

The sizeable heritability observed for tracheid length under control conditions echoes the results of Beaulieu (2003) for tracheid length in older white spruce trees. In particular, tracheid length is an important wood quality attribute because it has a significant impact on the properties of pulp and paper products, as well as fiber-based products such as wood-plastic composites and fiberboards (Beaulieu, 2003; Mvolo et al., 2015a; Mvolo et al., 2015b; Ouyang et al., 2018). Repeated drought conditions over long periods could therefore indirectly reduce the quality of wood for pulp production by resulting in a reduction in tracheid length. With repeated drought episodes over years, it could also lead to a decrease in wood uniformity, which could impact the physico-mechanical attributes of lumber products (Hassegawa et al., 2020; Soro et al., 2022). Further work will be necessary to determine how this trait should be considered on the long term. For wood quality, longer tracheids are generally desirable, however, the observed reduction of tracheid length as a response to drought stress may represent an important adaptive mechanism that may be key to seedling survival and growth (see above). Future work should investigate this trait under drought stress conditions at an older age and examine how it may affect survival to extreme drought. In particular, the genetic correlations between this trait and other key wood quality traits should also be assessed on the long term, if shorter tracheids are key to spruce survival under severe drought-stress conditions at the juvenile stage.

Spearman’s rank-order correlations showed no significant effects of drought-induced stress on the rankings of the clones for all traits, except for the number of pits per tracheid between the control and the severe drought-stress treatment (**Figure 6**). Thus, it appears that clones responded differently for this trait under severe drought conditions but not so much for the other wood anatomical and hydraulic conductivity traits. Indeed, the results indicate a relative stability in the ranks of clonal values for most of these attributes across the different treatments of this experiment. This important result indicates that juvenile selection for improved wood anatomical or hydraulic conductivity attributes for better resilience to moderate or severe drought conditions could be conducted under normal growing conditions. This would simplify trait assessment and allowing larger numbers of clones to be screened, resulting in potentially higher genetic gains in resilience to drought conditions, or allowing to increase genetic diversity in the selected multiclonal varieties.

## Conclusions

The exposure of young white spruce seedlings to different levels of drought-induced stress revealed different responses in terms of wood anatomy and xylem theoretical conductivity. Our results showed that drought stress had little impact on most physico-anatomical wood traits, but lead to the production of shorter tracheids. Considering the significant genetic control observed and relative stability of clonal ranks for most physioco-anatomical traits between the control and drought-induced treatments, these trends indicate that juvenile selection for physico-anatomical wood traits in the context of improved avoidance of drought effects could be valuable and performed under normal growing conditions of seedlings.

Our results showed a trade-off in the allocation of resources at the genetic level between tracheid length and diameter for all treatments. Since this trade-off was not observed at the phenotypic level, except control treatment, it can be assumed that there is a clear resource allocation strategy at the genetic level between these two aspects of tracheid development. This strategy may result in a physiological response that would limit the risk of embolism by reducing the length of the tracheids under drought conditions. It also suggests that when evaluating specific hydraulic conductivity in conifers, Hagen-Poiseuille formula should be amended to better consider this trait in conductivity estimates.

Given the significant values of broad-sense heritability for most hydraulic conductivity and wood anatomical traits investigated in this study, useful genetic variation seemed to exist among clones in relation to drought-stress response and resilience. Clones with shorter and larger tracheids had more apical growth under severe drought-stress conditions, which could be a useful attribute for young seedlings growing under competition in the field. Therefore, early selection should consider both of these tracheid attributes. At the more fundamental level, studies aiming at the direct assessment of hydraulic conductivity and embolism under drought-stress conditions should be conducted to determine the level of correlation between tracheid length and the permeability of the xylem in such young conifer trees. Lumen diameter was stable between control and drought-induced treatments with significant broad-sense heritability, so that it could also be considered in combination with tracheid size attributes to screen large numbers of white spruce seedlings and clones without having to induce drought-stress conditions. In the context of climate change, these selection schemes would allow for improved wood anatomical attributes conferring better resistance to moderate and severe drought-stress conditions at the young seedling stage.

## Data availability statement

The raw data supporting the conclusions of this article will be made available by the authors, without undue reservation.

## Author contributions

AS: Data curation, Formal analysis, Writing – original draft. PL: Conceptualization, Funding acquisition, Writing – review & editing. JR: Data curation, Writing – review & editing. SN: Formal analysis, Writing – review & editing. DP: Writing – review & editing. JB: Conceptualization, Funding acquisition, Writing – review & editing. AA: Conceptualization, Funding acquisition, Writing – review & editing.
