# Supplementary material for: The phenotypic and genetic effects of drought-induced stress on wood specific conductivity and anatomical properties in white spruce seedlings, and relationships with growth and wood density
Source: Front Plant Sci. 2023 Dec 22;14:1297314. doi: 10.3389/fpls.2023.1297314 (PMC10766765; doi:10.3389/fpls.2023.1297314)
Supplement: Supplementary file 1 [file Table_1.docx]

**Supplementary material for online-only publication**

To estimate genetic correlations between traits for each treatment, bivariate models were fit for all pairs of traits in ASReml-R using the following model:

$\left[ \begin{aligned} \boldsymbol{y}_{\boldsymbol{i}} \\ \boldsymbol{y}_{\boldsymbol{j}} \end{aligned} \right]=\boldsymbol{X\beta}+\boldsymbol{Z}_{\boldsymbol{1}}\boldsymbol{a}\boldsymbol{(}\boldsymbol{t}\boldsymbol{)}+\boldsymbol{Z}_{\boldsymbol{2}}\boldsymbol{b}\boldsymbol{(}\boldsymbol{t}\boldsymbol{)}+\boldsymbol{Z}_{\boldsymbol{3}}\boldsymbol{c}\boldsymbol{(}\boldsymbol{t}\boldsymbol{)}+\boldsymbol{e}$ (1)

where ***y_i_*** and ***y_j_*** are the stacked vectors of observations for traits 1 and trait 2; ***β*** represents the vector of fixed effects, including an overall mean for each trait; ***a(t)*** is the random additive genetic effect within trait, with ***a(t)***∼ *N*(**0**, ***V_a_*** ⊗ ***A***); ***b(t)*** is the random block effect for each trait and ***c(t)*** is the random clonal effect within trait, with ***c*** ∼ *N*(**0**, ***V_c_*** ⊗ ***I_c_***); and ***e*** is the error term, with ***e*** ∼ *N*(**0**, ***I_e_*** ⊗ ***V_r_***). ***A*** is the pedigree-based additive relationship matrix and ***I_c_*** and ***I_e_*** are a identity matrices of their proper dimension. The matrices ***V_a_***, ***V_c_*** and ***V_r_*** are 2 x 2 variance-covariance matrices as defined by the correlation of effects between traits (*r_a_*, *r_c_* and *r_e_*, respectively) and unique variances for each trait. The matrices ***X***, ***Z_1_***, ***Z_2_***, and ***Z_3_*** are incidence matrices of their corresponding effects. Phenotypic correlations between traits were calculated as:

$\hat{r}_{p}=\frac{\hat{r}_{a}\sqrt{\hat{\sigma}_{aⅈ}^{2}\hat{\sigma}_{aj}^{2}} + \hat{r}_{c}\sqrt{\hat{\sigma}_{cⅈ}^{2}\hat{\sigma}_{cj}^{2}} + \hat{r}_{e}\sqrt{\hat{\sigma}_{eⅈ}^{2}\hat{\sigma}_{ej}^{2}}}{\sqrt{( \hat{\sigma}_{aⅈ}^{2}+ \hat{\sigma}_{cⅈ}^{2} + \hat{\sigma}_{ei}^{2}) ( \hat{\sigma}_{aj}^{2}+ \hat{\sigma}_{cj}^{2} + \hat{\sigma}_{ej}^{2})}}$ (2)

where $\hat{\sigma}_{aⅈ}^{2}, \hat{\sigma}_{cⅈ}^{2}\mathrm{and} \hat{\sigma}_{eⅈ}^{2}$ are the estimated additive, clonal and residual variance of trait *i* (same for trait *j*), respectively. Genetic correlations were calculated for the combined additive and non-additive genetic variance as:

$$\hat{r}_{g}=\frac{\hat{r}_{a}\sqrt{\hat{\sigma}_{aⅈ}^{2}\hat{\sigma}_{aj}^{2}} + \hat{r}_{c}\sqrt{\hat{\sigma}_{cⅈ}^{2}\hat{\sigma}_{cj}^{2}}}{\sqrt{( \hat{\sigma}_{aⅈ}^{2}+ \hat{\sigma}_{cⅈ}^{2} ) ( \hat{\sigma}_{aj}^{2}+ \hat{\sigma}_{cj}^{2} )}}$$

The significance of the genetic correlation (*H_0_: r_g_ = 0; H_1_: r_g_ ≠ 0*) was tested by performing a likelihood-ratio test with two degrees of freedom between the full model in Equation ([1](https://onlinelibrary.wiley.com/doi/full/10.1111/eva.12823#eva12823-disp-0004)) and a reduced model assuming *r_a_ = 0* and  *r_c_ = 0* (i.e., a diagonal ***V_a_***  and ***V_c_*** matrix). The significance of the phenotypic correlation (*H_0_: r_p_ = 0; H_1_: r_p_ ≠ 0*) was tested by performing a likelihood-ratio test with three degrees of freedom between the full model in Equation ([1](https://onlinelibrary.wiley.com/doi/full/10.1111/eva.12823#eva12823-disp-0004)) and a reduced model assuming no correlation between traits (i.e., *r_a_= 0*, *r_c_ = 0*, and *r_e_ = 0*).

**Supplementary Table 1:** Matrices of phenotypic correlations between the different traits for the control, moderate and severe drought stress treatments. Standard errors of estimates are in parentheses. Significance levels: *, *P* < 0.05; ​ **, *P* < 0.01; ​***, *P* < 0.001.

| **Treatments** | **Traits** | **Lumen diameter** | **Tracheid length** | **Tracheid diameter** | **Number of pits per tracheid** | **Apical growth** | **Ring width** | **Wood density** | **Biomass index** |
| --- | --- | --- | --- | --- | --- | --- | --- | --- | --- |
| Control | Specific conductivity | 0.99 (0.00)*** | 0.22 (0.02)* | -0.10 (0.04)* | 0.08 (0.01)* | -0.04 (0.01)* | 0.26 (0.06)* | -0.50 (0.12)* | -0.34 (0.08)* |
|  | Lumen diameter |  | -0.21 (0.01)* | 0.03 (0.01)* | -0.18 (0.06)* | -0.21 (0.04)* | -0.16 (0.04)** | 0.03 (0.01) | -0.14 (0.06) |
|  | Tracheid length |  |  | -0.06 (0.01)* | 0.32 (0.07)* | -0.18 (0.01)* | 0.45 (0.20)* | 0.24 (0.05)* | 0.42 (0.13)* |
|  | Tracheid diameter |  |  |  | -0.01 (0.00) | 0.15 (0.05)* | -0.07 (0.01) | -0.11 (0.04)* | -0.04 (0.00) |
|  | Number of pits per tracheid |  |  |  |  | -0.09 (0.02)* | 0.06 (0.02) | 0.34 (0.15) | 0.21 (0.12) |
|  | Apical growth |  |  |  |  |  | 0.45 (0.13)** | 0.10 (0.02)** | 0.30 (0.07)** |
|  | Ring width |  |  |  |  |  |  | 0.17 (0.05)* | 0.45 (0.17)** |
|  | Wood density |  |  |  |  |  |  |  | 0.53 (0.12)** |
| Moderate | Specific conductivity | 0.99 (0.00)*** | 0.03 (0.01) | -0.28 (0.10)* | 0.01 (0.00) | -0.14 (0.01)* | 0.38 (0.10)* | -0.45 (0.18)* | -0.40 (0.10)* |
|  | Lumen diameter |  | -0.04 (0.01)* | 0.03 (0.01)* | 0.01 (0.00) | 0.00 (0.00) | -0.33 (0.10)* | -0.05 (0.01)* | -0.20 (0.09) |
|  | Tracheid length |  |  | 0.23 (0.14) | 0.35 (0.16) | -0.13 (0.02)* | 0.25 (0.07)* | 0.09 (0.01)* | 0.41 (0.14)* |
|  | Tracheid diameter |  |  |  | 0.07 (0.00) | 0.08 (0.03) | -0.21 (0.05)* | -0.14 (0.06) | -0.10 (0.03)* |
|  | Number of pits per tracheid |  |  |  |  | 0.27 (0.10)* | 0.21 (0.15) | 0.08 (0.01)* | 0.21 (0.01)* |
|  | Apical growth |  |  |  |  |  | 0.51 (0.20)* | 0.10 (0.02)** | 0.30 (0.07)** |
|  | Ring width |  |  |  |  |  |  | 0.16 (0.05)* | 0.40 (0.14)** |
|  | Wood density |  |  |  |  |  |  |  | 0.45 (0.23)* |
| Severe | Specific conductivity | 0.99 (0.00)*** | 0.10 (0.01)* | -0.28 (0.05)* | 0.14 (0.05)* | -0.09 (0.00)* | 0.11 (0.04)* | -0.47 (0.17)* | -0.13 (0.06)* |
|  | Lumen diameter |  | 0.00 (0.03) | 0.08 (0.02)* | 0.07 (0.00)* | -0.36 (0.10)* | -0.05 (0.01)* | 0.22 (0.01)* | -0.02 (0.01) |
|  | Tracheid length |  |  | 0.15 (0.05)* | 0.25 (0.10)* | -0.28 (0.10)* | 0.27 (0.12)* | 0.18 (0.05)* | 0.26 (0.12)* |
|  | Tracheid diameter |  |  |  | 0.03 (0.00) | 0.00 (0.01) | -0.19 (0.11) | 0.04 (0.01)* | -0.18 (0.06)* |
|  | Number of pits per tracheid |  |  |  |  | -0.31 (0.18) | 0.21 (0.11) | 0.28 (0.10)* | 0.22 (0.09)* |
|  | Apical growth |  |  |  |  |  | 0.63 (0.23)* | 0.01 (0.00) | 0.23 (0.06)* |
|  | Ring width |  |  |  |  |  |  | 0.05 (0.00)* | 0.18 (0.10)* |
|  | Wood density |  |  |  |  |  |  |  | 0.20 (0.10) |

**Supplementary Table 2:** Matrices of genotypic (genetic) correlations between the different traits for the control, moderate and severe drought stress treatments. Standard errors of estimates are in parentheses**.** Significance levels: *, *P* < 0.05; ​ **, *P* < 0.01; ​***, *P* < 0.001.

| **Treatments** | **Traits** | **Lumen diameter** | **Tracheid length** | **Tracheid diameter** | **Number of pits per tracheid** | **Apical growth** | **Ring width** | **Wood density** | **Biomass index** |
| --- | --- | --- | --- | --- | --- | --- | --- | --- | --- |
| Control | Specific conductivity | 0.99 (0.00)*** | 0.78 (0.21)* | -0.18 (0.45) | 0.28 (0.22) | -0.05 (0.37) | 0.86 (0.40)* | -0.84 (0.14)* | -0.93 (0.18)* |
|  | Lumen diameter |  | -0.24 (0.01)* | 0.80 (0.33)* | -0.26 (0.10)** | 0.50 (0.23)** | -0.60 (0.30) ** | -0.52 (0.35)** | -0.56 (0.21)** |
|  | Tracheid length |  |  | -0,55 (0.20)* | 0.59 (0.25)* | -0.25 (0.04)* | 0.80 (0.27)** | 0.82 (0.18)* | 0.99 (0.04)* |
|  | Tracheid diameter |  |  |  | -0.60 (0.30) | 0.80 (0.17)* | -0.61 (0.30)* | -0.82 (0.18)* | -0.69 (0.34) |
|  | Number of pits per tracheid |  |  |  |  | -0.10 (0.01) | 0.35 (0.10)* | 0.95 (0.12)* | 0.82 (0.33)* |
|  | Apical growth |  |  |  |  |  | -0.95 (0.21)** | 0.24 (0.10)* | 0.75 (0.32)* |
|  | Ring width |  |  |  |  |  |  | 0.85 (0.29)** | 0.85 (0.24)** |
|  | Wood density |  |  |  |  |  |  |  | 0.92 (0.20)** |
| Moderate | Specific conductivity | 0.95 (0.00)*** | 0.68 (0.10)* | -0.12 (0.13) | 0.20 (0.00)* | -0.01 (0.01) | 0.80 (0.41)* | -0.80 (0.18)* | -0.85 (0.23)* |
|  | Lumen diameter |  | 0.00 (0.00) | 0.70 (0.07)* | -0.59 (0.28)** | 0.58 (0.20)** | -0.57 (0.40)* | -0.55 (0.32)* | -0.70 (0.40) |
|  | Tracheid length |  |  | -0.57 (0.30)* | 0.39 (0.03)** | 0.08 (0.01)* | 0.41 (0.29)* | 0.17 (0.01)* | 0.78 (0.29)* |
|  | Tracheid diameter |  |  |  | -0.10 (0.01)* | 0.42 (0.18)* | -0.10 (0.01)* | -0.48 (0.23) | -0.55 (0.29) |
|  | Number of pits per tracheid |  |  |  |  | 0.75 (0.49) | 0.70 (0.30)* | 0.70 (0.37)* | 0.59 (0.38) |
|  | Apical growth |  |  |  |  |  | -0.15 (0.10)* | 0.14 (0.08) | 0.55 (0.10)* |
|  | Ring width |  |  |  |  |  |  | 0.58 (0.21)* | 0.86 (0.20)* |
|  | Wood density |  |  |  |  |  |  |  | 0.71 (0.24)* |
| Severe | Specific conductivity | 0.90 (0.00)*** | 0.65 (0.08)* | -0.10 (0.13) | 0.00 (0.00) | 0.00 (0.00) | 0.76 (0.41) | -0.77 (0.15)* | -0.82 (0.17)* |
|  | Lumen diameter |  | 0.49 (0.30) | 0.73 (0.07)* | -0.65 (0.29)* | 0.49 (0.21)* | -0.50 (0.43) | -0.70 (0.40) | -0.75 (0.45) |
|  | Tracheid length |  |  | -0.32 (0.10)* | 0.51 (0.05)** | -0.50 (0.29)* | 0.21 (0.07)* | 0.63 (0.28)* | 0.70 (0.20)* |
|  | Tracheid diameter |  |  |  | -0.50 (0.30) | 0.37 (0.10)* | -0.51 (0.15)* | -0.43 (0.13)* | -0.75 (0.25)* |
|  | Number of pits per tracheid |  |  |  |  | 0.77 (0.45) | 0.72 (0.27)* | 0.49 (0.27) | 0.71 (0.31)* |
|  | Apical growth |  |  |  |  |  | -0.15 (0.10) | 0.50 (0.20)* | 0.45 (0.01)** |
|  | Ring width |  |  |  |  |  |  | 0.58 (0.21)* | 0.49 (0.28) |
|  | Wood density |  |  |  |  |  |  |  | 0.65 (0.12)** |

**Supplementary Table 3:** Individual tree-level narrow sense ($\hat{h}^{2}$) et broad-sense ($\hat{H}^{2}$) heritability estimates for specific conductivity, lumen diameter, tracheid length, tracheid diameter and number of pits per tracheid. Standard errors of estimates are in parentheses.

| **Treatments** | **Traits** | $\hat{h}^{2}$ | | $\hat{H}^{2}$ |
| --- | --- | --- | --- | --- |
| Control | Specific conductivity | 0.20 (0.10) | 0.25 (0.15) | |
|  | Lumen diameter | 0.10 (0.01) | 0.13 (0.01) | |
|  | Tracheid length | 0.31 (0.21) | 0.44 (0.18) | |
|  | Tracheid diameter | 0.12 (0.02) | | 0.24 (0.10) |
|  | Number of pits per tracheid | 0.35 (0.27) | 0.54 (0.28) | |
| Moderate | Specific conductivity | 0.05 (0.00) | 0.17 (0.07) | |
|  | Lumen diameter | 0.13 (0.06) | 0.22 (0.14) | |
|  | Tracheid length | 0.10 (0.02) | 0.23 (0.10) | |
|  | Tracheid diameter | 0.00 (0.00) | 0.29 (0.10) | |
|  | Number of pits per tracheid | 0.00 (0.00) | 0.15 (0.13) | |
| Severe | Specific conductivity | 0.08 (0.00) | 0.22 (0.02) | |
|  | Lumen diameter | 0.38 (0.20) | 0.42 (0.18) | |
|  | Tracheid length | 0.03 (0.01) | 0.31 (0.12) | |
|  | Tracheid diameter | 0.00 (0.00) | 0.28 (0.10) | |
|  | Number of pits per tracheid | 0.12 (0.03) | 0.17 (0.10) | |
